# Low CD46 expression on activated CD4^+^ T cells predict improved Th1 cell reactivity to calcitriol in majority of patients with allergic eosinophilic asthma and healthy donors

**DOI:** 10.3389/falgy.2024.1462579

**Published:** 2024-09-24

**Authors:** Julie Stichova, Peter Slanina, Zita Chovancova, Jan Baros, Marek Litzman, Jiri Litzman, Marcela Vlkova

**Affiliations:** ^1^ Faculty of Medicine, Institute of Clinical Immunology and Allergology, Masaryk University, Brno, Czechia; ^2^ Institute of Clinical Immunology and Allergology, St. Anne's University Hospital, Brno, Czechia; ^3^ Department of Economics, Faculty of Business and Economics, Mendel University, Brno, Czechia

**Keywords:** allergic eosinophilic asthma, calcitriol, CD46, IFN-γ, IL-10

## Abstract

**Background:**

Previous research showed that the intracellular complement system, with CD46 as its central molecule, regulates the Th1 response associated with IFN-γ production and transition to a type 1 regulatory response (Tr1) characterized by IL-10 production. This transition can be influenced by a vitamin D (calcitriol), favouring a shift towards Tr1 cells and increased IL-10 production, as described in some autoimmune diseases.

**Objective:**

It is unknown whether calcitriol modulates CD46-induced Th1 response towards regulatory type 1 T cells (Tr1) in allergic eosinophilic asthma and its value in relation to reducing inflammatory response.

**Methods:**

CD4^+^ T cells from 58 patients with allergic eosinophilic asthma (AEA) and 49 healthy donors (HDs) were stimulated with αCD3/αCD46/IL-2 or αCD3/αCD46/IL-2/Calcitriol *in vitro* for 60 h and analyzed by flow cytometry. IFN-γ and IL-10 levels in cell culture supernatants were measured using ELISA.

**Results:**

CD4^+^ T cells from patients with AEA demonstrated elevated CD46 expression in both the non-activated state and under stimulation conditions with αCD3/αCD46/IL-2 or αCD3/αCD46/IL-2/Calcitriol. Moreover, CD46 expression in AEA patients fluctuated with the pollen season, showing a significant increase during period of low pollen exposure. Calcitriol further induced CD4^+^Tr1 cells from *in vitro* generated CD4^+^Th1 cells in both HDs and AEA patients. However, in both cohorts were individuals (HDs: 35/49, AEA: 40/58) who responded to calcitriol with a more pronounced regulatory response. The calcitriol-induced regulatory effect manifested by a stronger surface decrease of CD46 on activated CD4^+^ T cells (by 40% in HDs and by 26% in AEA), accompanied by a significant inhibition of IFN-γ and increased IL-10 production (by 31% in HDs and by 85% in AEA). These individuals were termed as the CD46D group. Contrary to this, calcitriol induced an increase in CD46 expression at the CD4^+^ T cell surface in a minor group of HDs (14/49), and AEA patients (18/58), who were termed as the CD46I group. In CD46I group, CD4^+^ T cells produced less IFN-γ in comparison with CD46D group (by 33% in HDs and by 43% in AEA) and were unable to upregulate IL-10 production following stimulation with αCD3/αCD46/IL-2/Calcitriol.

**Conclusion:**

Our results suggest the potential existence of a key for stratifying individuals suitable for calcitriol treatment in the context of low serum vitamin D levels. After validation in clinical studies, this key could be used as an adjunctive therapy not only for patients with allergic eosinophilic asthma, but also for other diseases.

## 1 Introduction

Allergic eosinophilic asthma (AEA) is a chronic inflammatory disease of the respiratory tract characterized by an excessively high immune response to common allergens with significant eosinophilic infiltration in the tissue of the respiratory tract leading to airway hyper-responsiveness (AHR) and obstruction (1). Therefore patients with AEA often experience persistent respiratory symptoms such as wheezing, coughing, shortness of breath, and chest tightness.

Although Th2 cells are considered a primary culprit in AEA pathophysiology, recent pieces of evidence suggest that Th1 cells may also contribute to AEA pathophysiology, thus confronting the paradigm that AEA is a result of Th2/Th1 imbalance and emphasizing the complex immune dynamics in AEA (2). However, more comprehensive data regarding the involvement of Th1 cells in AEA are still lacking, as well as information on the role of intracellular complement (also known as “complosome”) in CD4^+^ T cells in AEA (3).

The complosome critically regulates the differentiation of naive CD4^+^ T cells into effector Th1 cells producing high amounts of the pro-inflammatory interferon-gamma (IFN-γ) and the autocrine growth factor interleukin 2 (IL-2) following antigen encounter. Additionally, it regulates the transition of Th1 cells into type 1 regulatory T cells (Tr1), which produce IL-10, significantly contributing to halting an immune response (4). Most importantly, CD46 is considered the central regulatory molecule responsible for fine tuning the Th1 response in CD4^+^ T cells within the complosome system.

Membrane cofactor protein (CD46; MCP) is a receptor expressed on all nucleated cells (5). CD46 consists of four complement control repeats at the N-terminus, followed by a glycosylated region, a transmembrane segment and two cytoplasmic tails Cyt1 and Cyt2, which are co-expressed and can transmit signals (6). When a CD4^+^ T cell is activated via TCR, it starts to locally release C3b from intracellular C3 stores, which is generated by cathepsin L. Once translocated to the cell surface, C3b binds to CD46, leading to its activation (3). Subsequently, CD46 activation induces a Th1 phenotype with IFN-γ production in presence of low concentration of IL-2 during the early phase of the immune response. However, when IL-2 reaches a threshold concentration in the surrounding milieu during the late phase of the immune response, CD46-mediated signals induce inhibition of IL-2 production and simultaneously promote differentiation into Tr1 cells with potent production of IL-10 (5, 7).

Moreover, it has been shown that CD46 induced Tr1 differentiation can be boosted by vitamin D_3_ (its hormonal form, 1α,25-dihydroxyvitamin D_3_, also known as calcitriol), which strongly supports IL-10 production via the vitamin D receptor (VDR), which is expressed on the surface of activated CD4^+^ T cells (8–10). This particular ability of CD46 to promote IL-10 production, which can be enhanced by calcitriol is significant for developing therapeutic strategies for autoimmune and inflammatory diseases (5). It also present a potential strategy for treating asthma (11), as decreased IL-10 mRNA (12) and IL-10 levels in bronchoalveolar lavage or serum (13), together with defective CD4^+^CD25^+^ Treg cells (14), have been described in patients with asthma.

However, despite intensive research on the role of calcitriol in asthma, recent clinical trials provide unclear results regarding the effectiveness of calcitriol in asthma control (15, 16). Therefore, we decided to study CD46-induced Th1/Tr1 differentiation in CD4^+^ T cells *in vitro,* as well as the modulatory effect of calcitriol in a group of adult patients with AEA. We believe that new data regarding the role of Th1 cells in AEA along with the modulatory effect of calcitriol at the single-cell level could provide a clearer understanding of the immunopathology of AEA and may help clarify existing uncertainties about calcitriol's effect on asthma control.

## 2 Methods

### 2.1 Study participants

Overall, 58 adult patients with AEA and 49 healthy donors (HDs) were included in this study. The group of AEA patients consisted of 36 females and 22 males (19–62 years of age; median 42 years). AEA was diagnosed according to the GINA guidelines (17). All investigated patients with AEA had clinically relevant allergic symptoms, accompanied by exacerbation of asthmatic symptoms in the pollen season of spring flowering trees (mainly birch trees group), wild and cultivated grasses, and mugwort (Supplementary Figure S1). All patients had compensated asthma symptoms during the study. The patients with moderate (*n* = 37) to severe (*n* = 3) asthmatic symptoms were treated with inhaled corticosteroids (ICS) mostly in combination with long-acting beta agonists (LABA) throughout the year. Patients with mild (*n* = 18) seasonal asthmatic symptoms were treated only during their allergy pollen season. All AEA patients underwent two blood collections: the first during the active pollen season (high pollen period, HPP) from February to August in the Czech Republic, and the second outside of the active pollen season (low pollen period, LPP) from October to January. Those patients who were treated with oral corticosteroids at least 6 months before study initiation or who received allergen immunotherapy were excluded from the study.

HDs included 27 females and 22 males (22–76 years of age, median 30 years) with no clinical symptoms of allergy, also no laboratory signs of atopy (including raised serum levels of IgE, presence of specific IgE against most frequent allergens, elevated levels of ECP, or eosinophils in blood) were present. All AEA patients and HDs signed an informed consent form to participate in this study and their anonymity was preserved using methods approved by the Ethics Committee, approval number 34/2018.

### 2.2 Blood investigations

Total IgE levels were measured using the Immage-800 *(Beckman Coulter, Brea, CA, USA*), while specific IgE and ECP were assessed using the Immulite 2000 Xpi (*Siemens Healthineers AG, Forchheim, Germany*). The concentration of serum 25-OH Vitamin D was assessed using the Abbott Architect (*Abbott, Illinois, USA*). Relative and absolute counts of eosinophils were measured using a hematology analyzer Sysmex XN-3000 (*Sysmex Corporation, Kobe, Japan*).

### 2.3 CD4^+^ T cell isolation

Specimens of peripheral blood (10 ml) were collected into tubes with K_3_EDTA. Subsequently, peripheral blood mononuclear cells (PBMCs) were isolated using a standard Ficoll-Paque gradient centrifugation according to the manufacturer's instructions (*Amersham Pharmacia, Upsala, Sweden*) and cryopreserved at −80°C in freezing media containing fetal bovine serum (FBS; *HyClone, South Logan, UT, USA*) and 10% dimethyl sulfoxide (DMSO; *Sigma-Aldrich Chemie GmbH, Steinheim, Germany*). After collecting all specimens, PBMCs were thawed according to the protocol described by Kreher et al. (18) with an additional step of 30 min incubation with DNase I (10 μg/ml; *Roche Diagnostics GmbH, Mannheim, Germany*) to prevent the clumping of PBMCs after thawing. Next, CD4^+^ T cells were isolated from the PBMCs using the EasySep™ Human CD4^+^ T cell isolation kit (*STEMCELL Technologies, Inc., Vancouver, Canada*) by following the standard manufacturer procedure. Isolated CD4^+^ T cells were resuspended in a complete RPMI 1,640 medium supplemented with L-glutamine (2 mM), penicillin (50 U/ml), streptomycin (50 μg/ml) (*Sigma-Aldrich Chemie GmbH*), and 10% FBS and counted. The purity and viability of CD4^+^ T cells were measured by flow cytometry with results >93% and >95%, respectively.

### 2.4 CD4^+^ T cell culture

Isolated CD4^+^ T cells were cultured in complete RPMI 1,640 medium at a concentration of 7.5 × 10^5^ cells/ml in 96-well round bottom tissue culture plates (*Thermo Fisher Scientific, Waltham, MA, USA*). To trigger the activation of CD4^+^ T cells via CD46, we used a combination of αCD3/αCD46/IL-2, with both αCD3 and αCD46 immobilized in the wells. To evaluate the influence of calcitriol, we used the same stimuli combination (αCD3/αCD46/IL-2) in another well replenished with calcitriol (1α,25-Dihydroxyvitamin D_3_, also known as a hormonal form of Vitamin D_3_), as shown in Table 1.

**Table 1:** Work panel of used stimuli in cell culture.

Stimulus	Work concentration	Clone	Reference	Manufacturer
αCD3	10 μg/ml	HIT3a	300332	BioLegend
αCD46	5 μg/ml	MEM-258	11-342-C100	Exbio
IL-2	50 U/ml	x	200-02	PeproTech
Calcitriol	1 × 10^−7 ^M	x	D1530-10UG	Sigma Aldrich

### 2.5 Flow cytometry

CD4^+^ T cells were cultured with Brefeldin A for an additional 4 h, starting 60 h post-stimulation. While cell culture supernatants were collected and frozen at −20°C, CD4^+^ T cells were stained for flow cytometry (Table 2) according to the Affymetrix eBioscience intracellular antigens staining protocol A, using the Intracellular Fixation & Permeabilization Buffer Set (cat. no. 88-8824). Flow cytometry analysis was conducted on flow cytometer Navios EX (10 colors, 3 lasers, *Beckman Coulter, Inc. Miami, FL, USA*), following the gating strategy outlined in Supplementary Figure S2 and the data were analyzed using the Kaluza software (*Beckman Coulter, Brea, CA, USA*).

**Table 2:** Work panel used and monoclonal antibody/dye information.

Antigen	Fluorochrome	Clone	Reference	Manufacturer
CD4	PerCP-Cy5.5	SK3	332772	Becton Dickinson
CD46	AF700	MEM-258	A7-342-T100	Exbio
IFN-γ	BV421	4SB3	B336876	BioLegend
IL-10	PE	JES3-19F1	B285627	BioLegend
Aqua fluorescent reactive dye	Live/Dead™	x	L34957 A	Invitrogen
CD25	PE-Cy7	B61498	A52882	Beckman Coulter
Ki-67	APC	Ki-67	B282019	BioLegend

AF700, alexa fluor 700; BV421, brilliant violet 421; PE, phycoerythrin; PE-Cy7, phycoerythrin cyanin 7; PerCP-Cy5.5, Peridinin-Chlorophyll-Protein Cyanine® 5.5; APC, allophycocyanin.

### 2.6 ELISA

CD4^+^ T cell supernatants (SNs) were collected after 60 h of stimulation and frozen at −20°C until measurement. IFN-γ and IL-10 levels in cell culture SNs were measured using the IFN-γ ELISA MAX™ Deluxe set and Human IL-10 ELISA MAX™ Deluxe set (both from *BioLegend, San Diego, CA, USA*) with appropriate dilution and analytical sensitivities of 2 and 4 pg/ml, respectively. The concentration of sIL-2RA (soluble IL-2R α chain) in cell culture SNs was assessed using the human IL-2RA ELISA Kit (*Thermo Fisher Scientific*, *Waltham, MA, USA*) with analytical sensitivity of 15 pg/ml. Soluble CD46 (sCD46) was assessed in plasma samples using the Human MCP/CD46 (Membrane Cofactor Protein) ELISA Kit (*Elabscience Biotechnology Inc. Houston, TX, USA*) with analytical sensitivity of 4.69 pg/ml.

### 2.7 Statistical analysis

Statistical evaluation of the results was performed using GraphPad Prism 5 (*GraphPad Software Inc.*). First, data were tested for normality using the Shapiro–Wilk test. Next, data were analyzed using the non-parametric Wilcoxon sign-rank test (for paired data) and Mann–Whitney *U*-test (for unpaired data) or the Kruskal–Wallis test with Dunn's correction for multiple comparisons where appropriate. Correlation analysis was performed using non-parametric Spearman correlation coefficient where appropriate. Statistically significant *p*-values were represented as symbols in graphs and were used as follows; *p < 0.05, **p < 0.01, ***p < 0.001, ns, non-significant.

## 3 Results

### 3.1 CD46 expression is increased in non-activated CD4^+^ T cells from patients with allergic asthma in low pollen period

**Figure 1:**
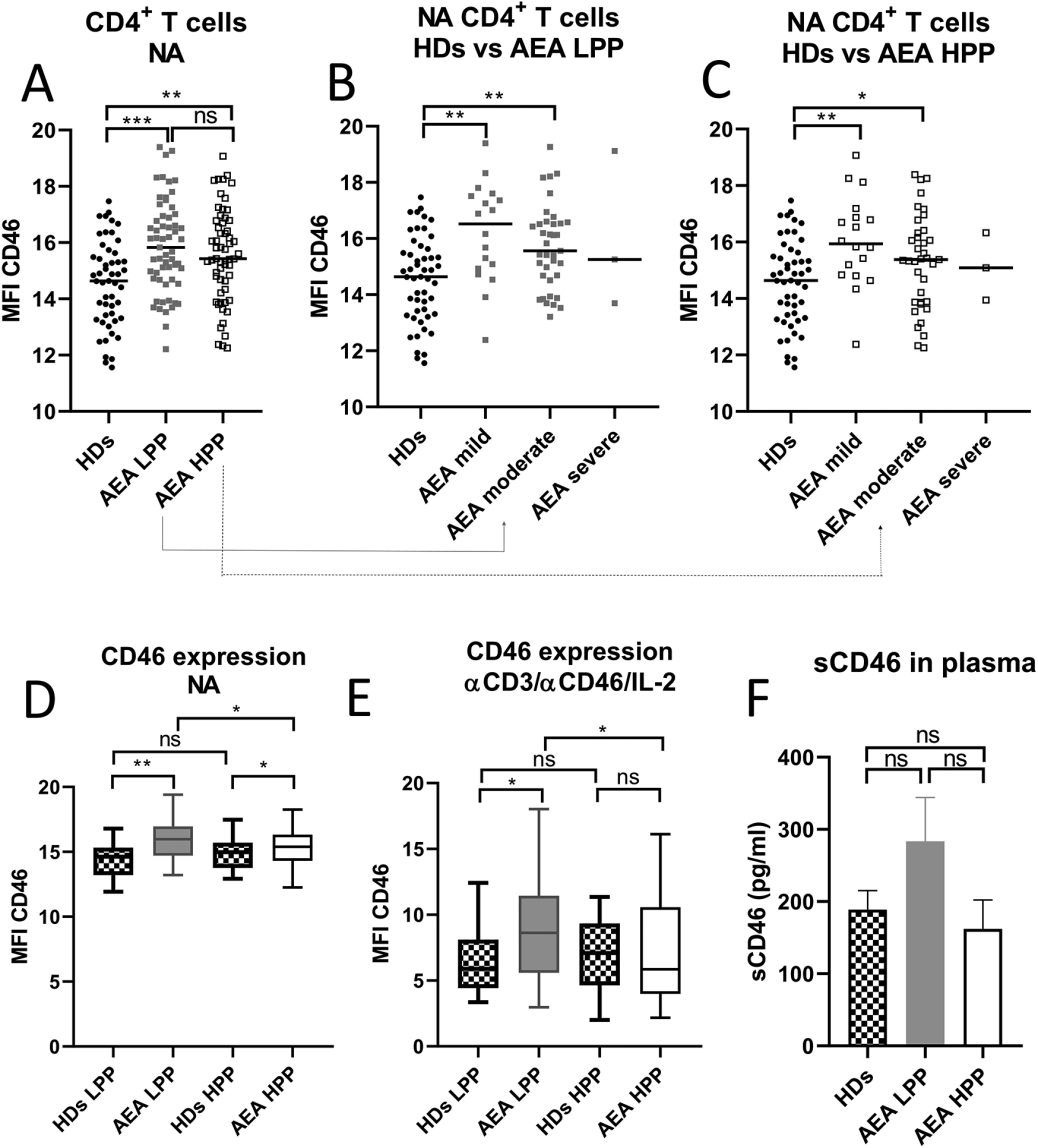
AEA patient's non-activated CD4^+^ T cells show increased surface expression of CD46, which is dependent on pollen season. Isolated CD4^+^ T cells from 49 HDs and 58 patients with AEA were analyzed non-activated (NA). The surface expression of CD46 on NA CD4^+^ T cells was assessed in HDs, AEA in LPP and HPP **(A)** as well as in HDs vs. AEA based on disease severity in LPP **(B)** and HPP **(C)**. Data were measured by flow cytometry and are presented as median fluorescence intensity (MFI) in graphs **(A–C)**. Horizontal bars represent the median. Next, the surface expression of CD46 was analyzed in HDs and AEA CD4^+^ T cells under both non-activated (NA) conditions **(D)** and after stimulation with αCD3 (10 μg/ml), αCD46 (5 μg/ml) mAbs and a high dose of IL-2 (50 U/ml), depending on LPP and HPP **(E)**. Concentration of sCD46 was assessed in plasma samples using ELISA in 40 HDs and 40 AEA patients in both LPP and HPP **(F)**. Data are presented as a median +95% CI in graphs **(D)**, **(E)** and **(F)**. Statistical analysis was performed using the unpaired Mann–Whitney *U*-test for two-group comparisons, and the Kruskal–Wallis test with Dunn's correction for three or more comparisons within a single graph where appropriate; ns (not significant), *p ≤ 0.05, **p ≤ 0.01, ***p ≤ 0.001. HDs, healthy donors; AEA, allergic eosinophilic asthma; NA, non-activated; LPP, low pollen period; HPP, high pollen period; MFI, median fluorescence intensity; CI, confidence interval.

Since CD46 is known as a key regulator of complosome-induced Th1 response, which is shed from the cell surface upon activation, we initially evaluated its expression on non-activated (NA) CD4^+^ T cells in HDs and AEA patients. Our observations revealed significantly increased expression of CD46 on CD4^+^ T cells from AEA patients (Figure 1A), with prominent elevation in mild and moderate asthma regardless of the allergy season (Figures 1B,C). However, due to the small number of participants, we were unable to assess CD46 expression on CD4^+^ T cells in patients with severe asthma (*n* = 3). Next, we investigated, whether CD46 expression remains consistent during the low pollen period (LPP) and the high pollen period (HPP) in AEA. Whereas CD46 expression was comparable between both periods in HDs as expected (Figures 1D,E), CD46 expression varied in AEA patients with significantly increased CD46 on NA CD4^+^ T cells from AEA patients in LPP (*p* < 0.0494) (Figure 1D) and also after αCD3/αCD46/IL-2 stimulation (*p* = 0.0432) (Figure 1E). Interestingly, CD4^+^ T cells from AEA patients in LPP exhibited a significantly less efficient decrease in CD46 expression after αCD3/αCD46/IL-2 stimulation compared to HDs (*p* < 0.0450). We further noticed that the increased CD46 expression on non-activated CD4^+^ T cells in AEA patients is likely not due to defective shedding from the cell surface, since plasma levels of the soluble form of CD46 were comparable to those in HDs (Figure 1F).

**Figure 2:**
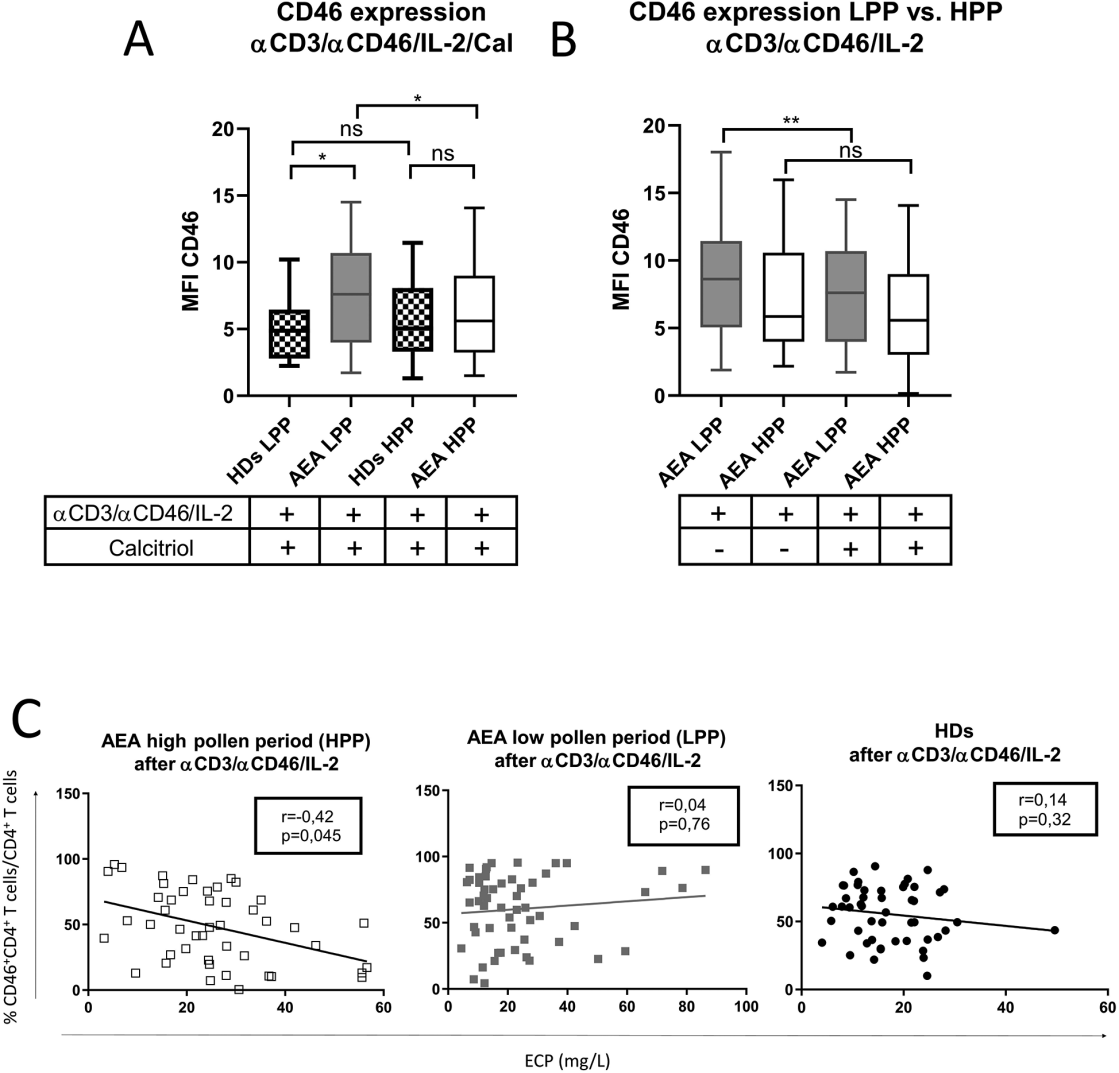
AEA patient's CD4^+^ T cells show increased expression of CD46 after stimulation in low pollen period. CD4^+^ T cells from 49 HDs and 58 patients with AEA were cultured with a mixture of αCD3 (10 μg/ml), αCD46 (5 μg/ml) mAbs and high dose of IL-2 (50 U/ml) (αCD3/αCD46/IL-2) for 60 h. **(A)** Surface expression of CD46 on CD4^+^ T cells was compared in both HDs and AEA patients in dependence of LPP and HPP, as well as between AEA patients in dependence of LPP and HPP **(B)**. Serum ECP levels were correlated with percentage of CD46^+^CD4^+^ T cells after αCD3/αCD46/IL-2 stimulation among HDs, AEA LPP and AEA HPP **(C)**. Surface expression of CD46 was measured by flow cytometry and is presented as median fluorescence intensity (MFI). Statistical analysis of CD46 expression was performed using Kruskal–Wallis test with Dunn's correction for multiple comparisons all vs. all. Correlation analysis was performed using non-parametric Spearman correlation coefficient; ns (not significant), *p ≤ 0.05, **p ≤ 0.01. HDs, healthy donors; AEA, allergic eosinophilic asthma; mAbs, monoclonal antibodies; LPP, low pollen period; HPP, high pollen period; ECP, eosinophilic cationic protein; MFI, median fluorescence intensity.

Increased CD46 expression was also observed after *in vitro* stimulation of CD4^+^ T cells from AEA patients with αCD3/αCD46/IL-2/Cal in LPP (*p* = 0.0328), whereas AEA in HPP showed normal expression of CD46 when compared to HDs (Figure 2A). Although calcitriol did not fully normalize CD46 downregulation in AEA LPP, it did partially reduce CD46 expression (*p* = 0.0095) (Figure 2B). Hence, we were further interested in whether the differences in CD46 expression might be specifically related to HPP, which is characterized by a more prominent Th2 response. To verify this assumption, we assessed the serum concentration of total IgE, eosinophilic cationic protein (ECP), the percentage of eosinophils and fractional exhaled nitric oxide (FeNO), all of which were increased in HPP AEA (*p* < 0.04) (Supplementary Figure S3). Then we also correlated the serum concentration of ECP with percentage of CD46^+^CD4^+^ T cells after αCD3/αCD46/IL-2 stimulation *in vitro*. We discovered a significant negative correlation between serum ECP levels and %CD46^+^CD4^+^ T cells (*p* = 0.045) in HPP AEA (Figure 2C). However, this correlation was absent in LPP AEA. Considering the potential impact of a more pronounced Th2 response on the Th1 reactivity of circulating CD4^+^ T cells in patients with AEA in HPP, we primarily focused on the Th1/Tr1 differentiation of CD4^+^ T cells from AEA patients during the LPP period.

### 3.2 CD4^+^ T cells show differences in CD46 expression after calcitriol stimulation in both healthy donors and patients with allergic asthma in low pollen period

**Figure 3:**
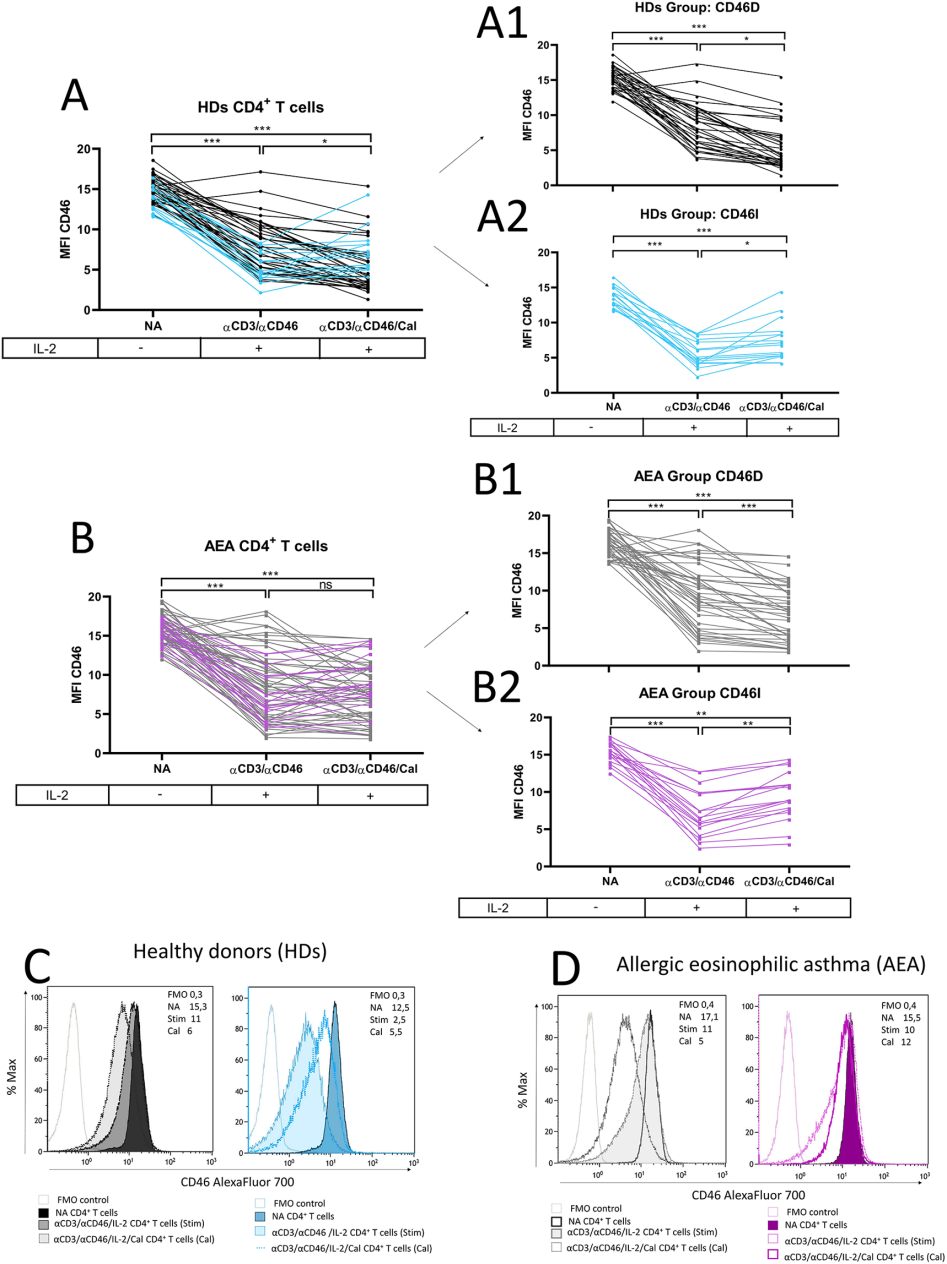
CD4^+^ T cells differ in CD46 downregulation after calcitriol co-stimulation in both HDs and AEA patients. CD4^+^ T cells from 49 HDs and 58 patients with AEA were cultured without stimuli (NA) or with a mixture of αCD3 (10 μg/ml), αCD46 (5 μg/ml) mAbs and high dose of IL-2 (50 U/ml) (αCD3/αCD46) for 60 h alone or with calcitriol (1 × 10^−7 ^M). The surface expression of CD46 was analyzed before/after calcitriol stimulation in HDs **(A)** and AEA patients in LPP **(B)**. Subsequently, two different groups were identified based on CD46 expression after calcitriol stimulation based on following formula: *x* = MFI CD46 (αCD3/αCD46/IL-2/Calcitriol)—MFI CD46 (αCD3/αCD46/IL-2). When *x* < 0 = Group CD46D (Decrease) **(A1,B1)**, when *x* > 0 = Group CD46I (Increase) **(A2,B2)**. Representative histograms depicts surface CD46 expression on NA or activated CD4^+^ T cells in groups CD46D/CD46I in HDs **(C)** and AEA patients **(D)**. Statistical analysis was performed using Kruskal–Wallis test with Dunn's correction for multiple comparisons all vs. all; ns (not significant), *p ≤ 0.05, **p ≤ 0.01, ***p ≤ 0.001. HDs, healthy donors; AEA, allergic eosinophilic asthma; NA, non-activated; mAbs, monoclonal antibodies; LPP, low pollen period; CD46D, group where CD46 expression on CD4^+^ T cells decrease after calcitriol stimulation; CD46I, group where CD46 expression on CD4^+^ T cells increase after calcitriol stimulation.

Since recent meta-analyses from clinical trials (15, 16) where patients with AEA were treated with vitamin D_3_ remain inconclusive, we were curious whether patients show a consistent pattern of CD46 downregulation after CD4^+^ T cell stimulation with or without calcitriol *in vitro*. We observed CD46 downregulation after αCD3/αCD46/IL-2 stimulation with calcitriol on HDs CD4^+^ T cells *in vitro* as described previously (19) (Figure 3A). Furthermore, we documented that CD46 downregulation occurs not only in CD4^+^ T cells phenotypically classified as CD46-negative, but also in CD46-positive CD4^+^ T cells (Supplementary Figure S4). This result indicates that stimulation affects CD46 expression across the entire CD4^+^ T cell population, not just the CD4^+^CD46^−^ subset. We were additionally able to distinguish two different patterns of CD46 expression in both HDs and AEA. Whereas the first group showed decreased CD46 [HDs *n* = 35 (71%), AEA *n* = 40 (69%), (Figures 3A1,B1)], the second group manifested by increased CD46 after calcitriol stimulation [HDs *n* = 14 (29%), AEA *n* = 18 (31%) (Figures 3A2,B2)]. These groups were termed as CD46D (Decrease) and CD46I (Increase), respectively, and were analyzed separately according to the following formula:

x=MFICD46(αCD3/αCD46/IL-2/Calcitriol)−MFICD46(αCD3/αCD46/IL-2)

Whenx<0=GroupCD46D

Whenx>0=GroupCD46I

The representative histograms of CD46 expression in HDs and AEA CD46D and CD46I groups are shown in Figures 3C,D.

### 3.3 The CD46D group of AEA patients shows dysregulation of CD46 following *in vitro* calcitriol stimulation during the low pollen period, independent of serum vitamin D levels

**Figure 4:**
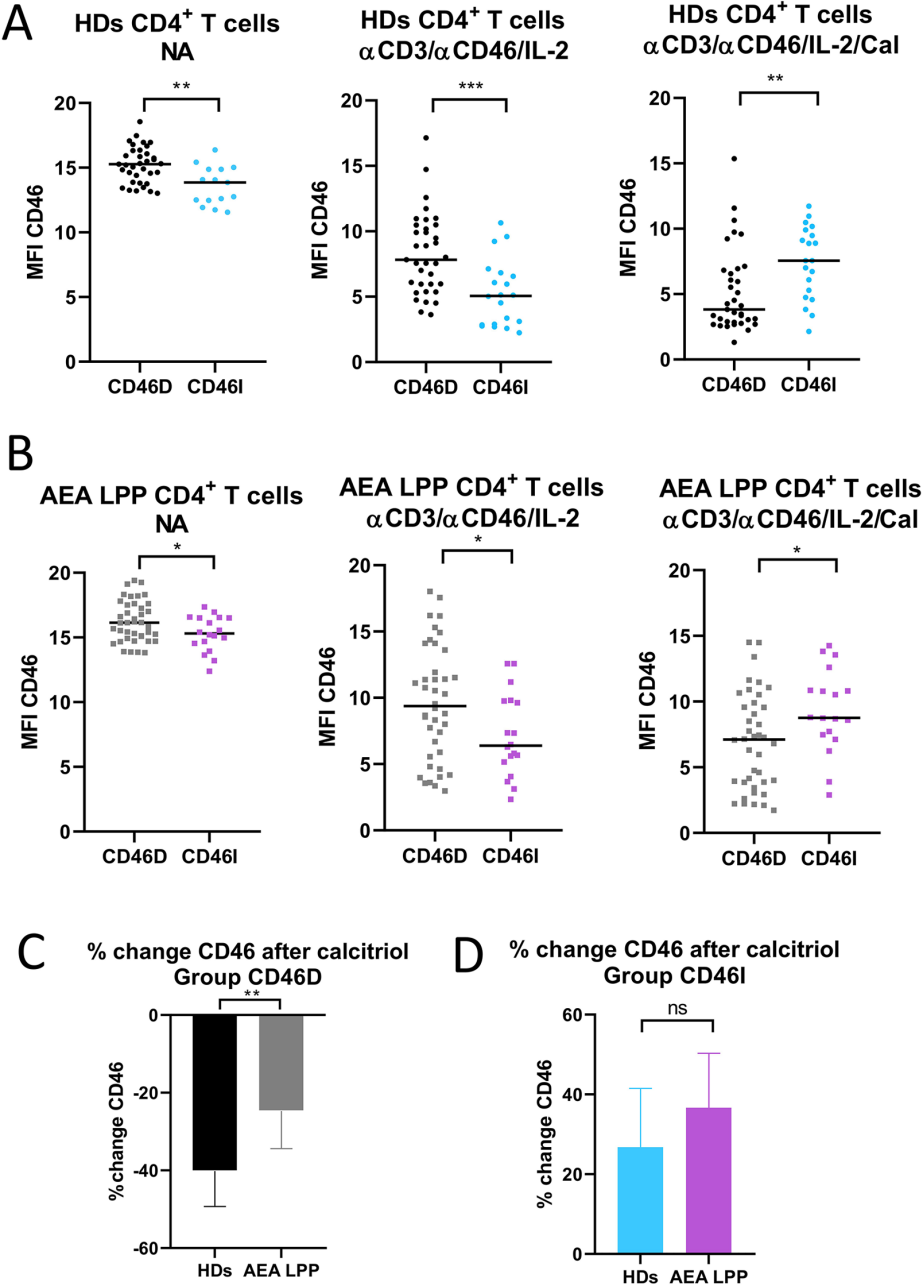
CD46D and CD46I groups of HDs and AEA patients differ in CD46 expression on both non-activated and stimulated CD4^+^ T cells. CD4^+^ T cells from 49 HDs and 58 patients with AEA were cultured without stimuli (NA) or with a mixture of αCD3 (10 μg/ml), αCD46 (5 μg/ml) mAbs and high dose of IL-2 (50 U/ml) (αCD3/αCD46/IL-2) and with calcitriol (1 × 10^−7 ^M) (αCD3/αCD46/IL-2/Cal). Surface expression of CD46 was measured in both groups CD46D/CD46I from HDs **(A)**, as well as from AEA patients **(B)**. Subsequently, percentage of CD46 downregulation after αCD3/αCD46/IL-2/Calcitriol stimulation was assessed in the group CD46D from HDs and AEA patients **(C)**. Identically, percentage of CD46 upregulation after αCD3/αCD46/IL-2/Calcitriol stimulation was assessed in the CD46I group from HDs and AEA patients **(D)**. The surface expression of CD46 was measured by flow cytometry and is presented as median fluorescence intensity (MFI) in graphs **(A)** and **(B)**. Graphs **(C)** and **(D)** are depicted as median + 95% CI. Statistical analysis was performed using the non-parametric Mann–Whitney *U* test; ns (not significant), *p ≤ 0.05, **p ≤ 0.01, ***p ≤ 0.001. HDs (healthy donors), AEA, allergic eosinophilic asthma; NA, non-activated; mAbs, monoclonal antibodies; CD46D, group where CD46 expression on CD4^+^ T cells decrease after calcitriol stimulation; CD46I, group where CD46 expression on CD4^+^ T cells increase after calcitriol stimulation; MFI, median fluorescence intensity; CI, confidence interval.

**Figure 5:**
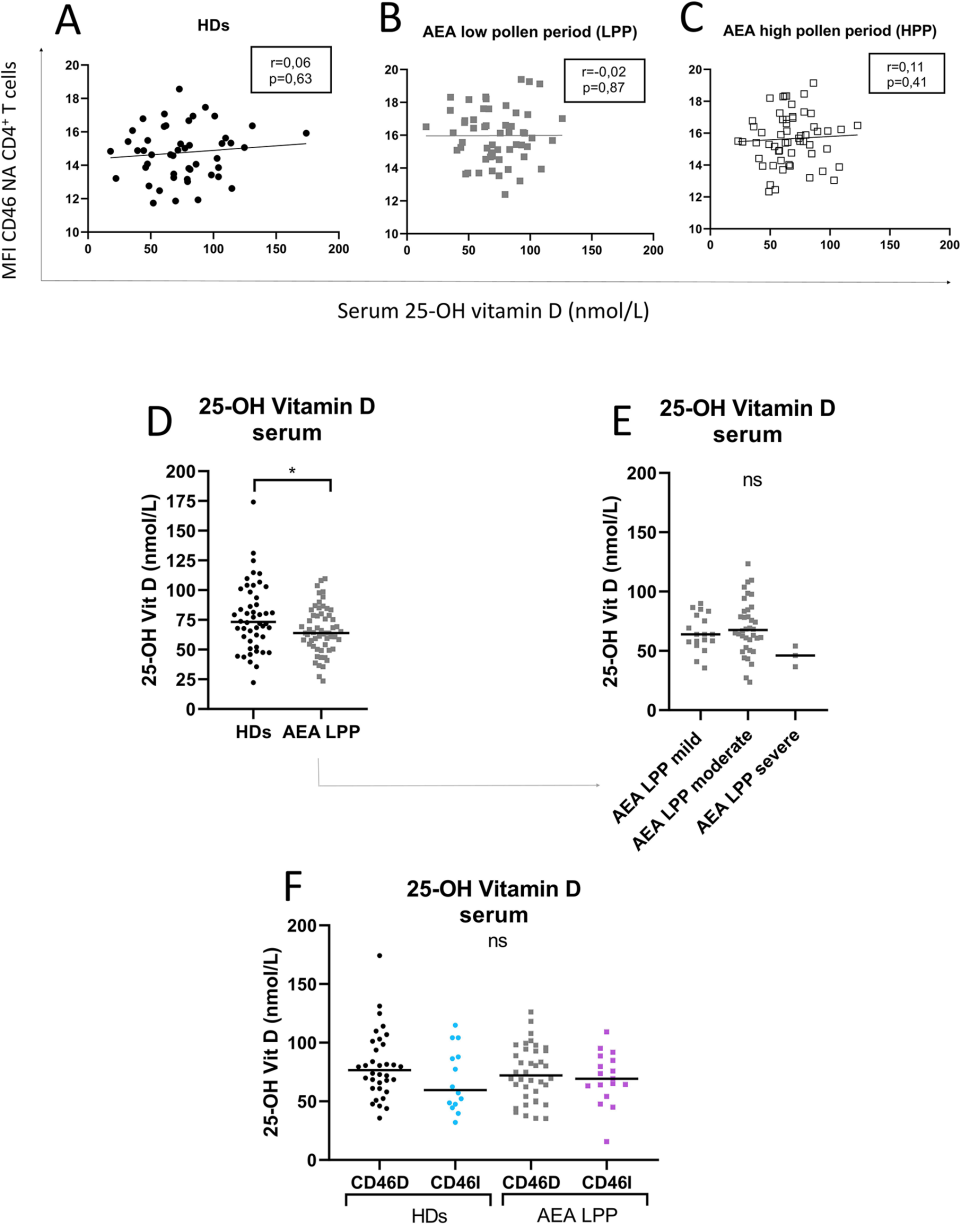
Increased CD46 expression on CD4^+^ T cells from both HDs and AEA patients is independent of serum 25-OH vitamin D concentration. To clarify the relationship between the surface expression of CD46 on NA CD4^+^ T cells and serum levels of 25-OH vitamin D, these parameters were correlated in both HDs (*n* = 49) **(A)** and AEA patients in LPP (*n* = 58) **(B)**, as well as in HPP **(C)**. Next, serum levels of 25-OH vitamin D were compared between HDs and AEA **(D)** as well as in dependence on asthma severity **(E)**. The serum concentration of 25-OH vitamin D was also compared after dividing HDs and AEA patients into CD46D/CD46I groups **(F)**. Correlation analysis was performed using the non-parametric Spearman correlation coefficient, comparison of serum 25-OH vitamin D levels between HDs and AEA was performed using the non-parametric Mann–Whitney *U*-test, while the Kruskal–Wallis test with Dunn's correction was used for multiple comparisons all vs. all; ns (not significant), *p ≤ 0.05. HDs, healthy donors; AEA, allergic eosinophilic asthma; NA, non-activated; CD46D, group where CD46 expression on CD4^+^ T cells decrease after calcitriol co-stimulation; CD46I, group where CD46 expression on CD4^+^ T cells increase after calcitriol co-stimulation.

Our next goal was to investigate, whether CD46 expression differs between the CD46D and CD46I groups in both HDs and AEA. Although CD46 was expressed more on NA CD4^+^ T cells in the CD46D group in both HDs (Figure 4A) and AEA (Figure 4B), after αCD3/αCD46/IL-2 stimulation with calcitriol *in vitro*, CD4^+^ T cells from HDs reduced CD46 by 40%, whereas those from AEA reduced it only by 26% (*p* = 0.0054) (Figure 4C). Conversely, CD4^+^ T cells from the CD46I group increased CD46 expression by 27% in HDs and by 37% in AEA after stimulation with calcitriol and αCD3/αCD46/IL-2. Despite AEA patients in the CD46I group had increased expression of CD46 after calcitriol by 10%, the results were not significant (*p* = 0.11) (Figure 4D). Furthermore, CD46 expression on NA CD4^+^ T cells did not correlate with serum concentrations of 25-OH vitamin D in HDs (*p* = 0.63) (Figure 5A), AEA LPP (*p* = 0.87) (Figure 5B), and AEA HPP (*p* = 0.41) (Figure 5C). Although AEA patients had a decreased serum concentration of 25-OH vitamin D (*p* = 0.04) (Figure 5D), it was independent of asthma severity (Figure 5E), CD46D/CD46I groups stratification (Figure 5F) as well as the pollen period (*p* > 0.05).

### 3.4 CD4^+^ T cells from AEA patients exhibit increased CD25, which is further promoted by calcitriol

**Figure 6:**
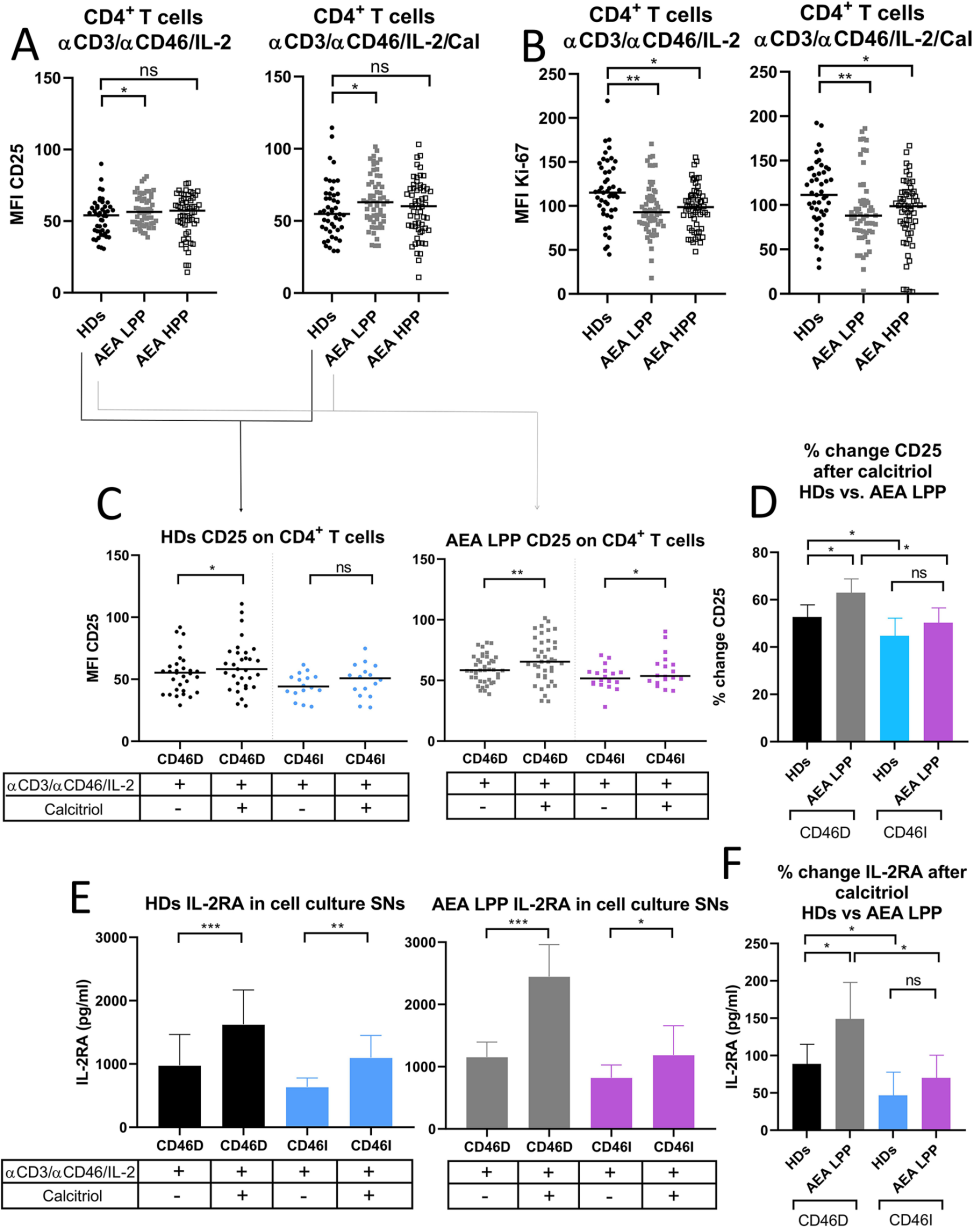
AEA patients in low pollen period show increased CD25 expression on stimulated CD4^+^ T cells, which is further promoted by calcitriol. CD4^+^ T cells from 49 HDs and 58 patients with AEA were cultured with a mixture of αCD3 (10 μg/ml), αCD46 (5 μg/ml) mAbs and high dose of IL-2 (50 U/ml) (αCD3/αCD46/IL-2) or with calcitriol (1 × 10^−7 ^M) (αCD3/αCD46/IL-2/Cal) for 60 h. **(A)** Surface expression of CD25 (IL-2 receptor α chain) and proliferation **(B)** were analyzed by flow cytometry before/after calcitriol stimulation with αCD3/αCD46/IL-2 in HDs and AEA patients in LPP and HPP groups using the non-parametric Mann–Whitney *U*-test. The data are presented as median intensity fluorescence (MFI), where horizontal bars indicate the median. Based on the CD46 dynamics after calcitriol stimulation, HDs and AEA patients were divided into groups CD46D/CD46I and surface expression of CD25 was analyzed separately. **(C)** To better understand the effect of calcitriol on CD25 expression in CD4^+^ T cells between HDs and AEA patients, we expressed the results as the percentage of upregulation after stimulation. Next, sIL-2RA levels were assessed in cell culture SNs, with HDs and AEA LPP patients divided into CD46D/CD46I groups **(E)** and are presented as a median +95% CI. Data from panels **(C)** and **(E)** were analyzed using the Wilcoxon-sign rank test and data from graphs **(D)** and **(F)** using the Kruskal–Wallis test with Dunn's correction; ns (not significant), *p ≤ 0.05. HDs, healthy donors; AEA, allergic eosinophilic asthma; mAbs, monoclonal antibodies; LPP, low pollen period; sIL-2RA, soluble IL-2 receptor α chain; CD46D, group where CD46 expression on CD4^+^ T cells decrease after calcitriol stimulation; CD46I, group where CD46 expression on CD4^+^ T cells increase after calcitriol stimulation; MFI, median fluorescence intensity; CI, confidence interval.

The CD46 pathway is modulated not only by crosstalk with VDR-calcitriol signaling but also by signals from the IL-2 receptor (IL-2R) in activated CD4^+^ T cells. Therefore, we assessed surface expression of IL-2R (CD25) on CD4^+^ T cells from HDs and AEA patients in both LPP and HPP. We observed that AEA patients in LPP had increased expression of CD25 on CD4^+^ T cells stimulated with or without calcitriol regardless of pollen season (Figure 6A). However, despite increased CD25 expression, CD4^+^ T cells from AEA LPP patients showed significantly reduced proliferation under αCD3/αCD46/IL-2 conditions, as well as after calcitriol supplementation, which had little to no effect (Figure 6B). We also found no difference in proliferation between CD46D and CD46I groups in both HDs and AEA patients.

Next, we analyzed CD25 expression on CD4^+^ T cells before and after calcitriol stimulation respecting stratification of HDs and AEA patients in LLP into CD46D and CD46I groups. We observed that calcitriol significantly increased CD25 expression on CD4^+^ T cells in HDs CD46D group (*p* = 0.0174) and even more so in the AEA LPP CD46D group (*p* = 0.0015) and with a weaker effect in the CD46I group (*p* = 0.048) (Figure 6C). However, when we analyzed the expression of CD25 as the percentage of positivity, we observed no significant difference.

However, whereas HDs in the CD46D group increased CD25 expression by 52%, AEA patients from the CD46D group were able to upregulate CD25 on CD4^+^ T cells by 63% after calcitriol stimulation (*p* = 0.035) (Figure 6D). Similar to surface CD25, IL-2RA was shed from the cell surface after calcitriol stimulation more in the HDs CD46D group (*p* < 0.0001, median 1,222 pg/ml) and even more so in the AEA LPP CD46D group (*p* < 0.0001, median 2,144 pg/ml). Calcitriol stimulation had a weaker effect in both the HDs CD46I group (*p* = 0.007, median 964 pg/ml) as well as the AEA LPP (*p* = 0.0487, median 1,015 pg/ml) (Figure 6E). When calculating the percentage change in IL-2RA in AEA LPP patients, we observed that calcitriol promoted IL-2RA shedding from activated CD4^+^ T cells by 148% in the CD46D group compared to HDs, whereas in the CD46I group, the increase was only by 70% (Figure 6F). We observed increased IL-2RA in cell culture SNs from AEA patients in comparison to HDs, generally independent of the pollen period (Supplementary Figure S3B).

### 3.5 CD46D and CD46I groups significantly differ in IFN-γ and IL-10 production in HDs and patients with AEA show increased IFN-γ production by activated CD4^+^ T cells, which can be restored by calcitriol *in vitro*

**Figure 7:**
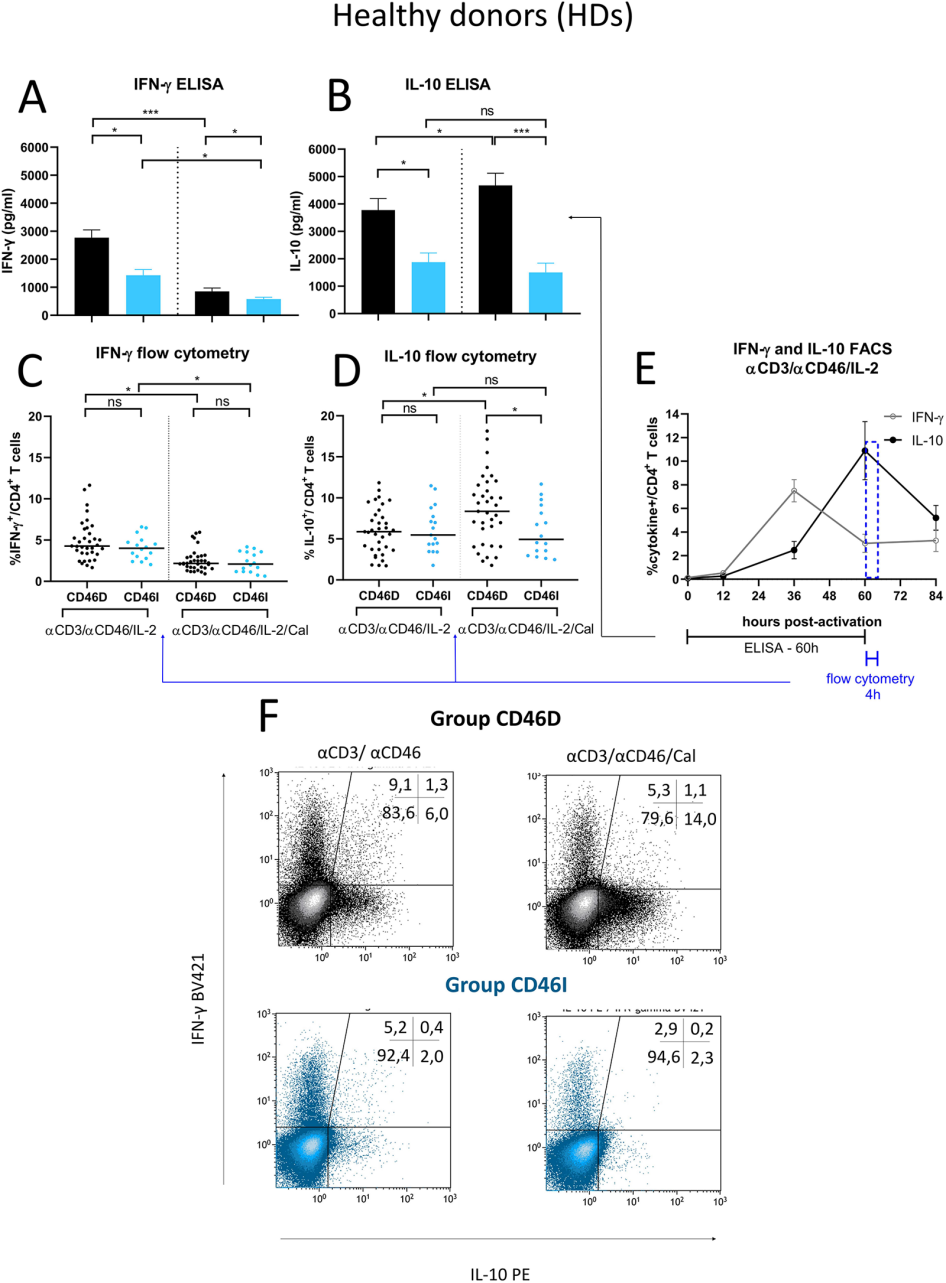
Stimulated CD4^+^ T cells from CD46D group produce significantly more IFN-γ and IL-10 in HDs and react to calcitriol co-stimulation with downregulation of IFN-γ and upregulation of IL-10. CD4^+^ T cells from 49 HDs were cultured with a mixture of αCD3 (10 μg/ml), αCD46 (5 μg/ml) mAbs and high dose of IL-2 (50 U/ml) (αCD3/αCD46/IL-2) or with calcitriol (1 × 10^−7 ^M) (αCD3/αCD46/IL-2/Cal) for 60 h. The concentrations of IFN-γ **(A)** and IL-10 **(B)** were assessed using ELISA and analyzed in accordance with the division of HDs into CD46D/CD46I groups. The results are presented as the median +95% CI. Similarly, the percentages of IFN-γ^+^CD4^+^ T cells **(C)** and IL-10^+^CD4^+^ T cells **(D)** obtained from flow cytometry were analyzed, with the horizontal bar representing the median. Data were analyzed using the Kruskal–Wallis test with Dunn's correction for multiple comparisons all vs. all. **(E)** The timeline showing IFN-γ and IL-10 production by αCD3/αCD46/IL-2-stimulated CD4^+^ T cells at 0, 12, 36, 60 and 84 h of incubation is based on data from three healthy donors and is presented as the median + 95% confidence interval. **(F)** Representative dot-plots depict differences in IFN-γ and IL-10 production by activated CD4^+^ T cells respecting the division into the groups CD46D/CD46I. The data from flow cytometry were obtained from CD4^+^ T cells treated with Brefeldin A during the final 4 h of stimulation and were gated from 90.000 CD4^+^ T cells. The numbers in the top right corner show percentage of CD4^+^ T cells in each quadrant. The data are representatives of 107 individual samples performed in 18 series. HDs, healthy donors; mAbs, monoclonal antibodies; CD46D, group where CD46 expression on CD4^+^ T cells decrease after calcitriol stimulation; CD46I, group where CD46 expression on CD4^+^ T cells increase after calcitriol stimulation.

Since CD46D/CD46I group stratification reflects different responses of surface CD46 on CD4^+^ T cells to stimulation with αCD3/αCD46/IL-2/Cal, we were interested whether they differ in the production of cytokines. We assessed pro-inflammatory cytokine IFN-γ, which is produced by effector Th1 cells, and IL-10, that is produced by various cell types such as CD4^+^CD25^+^ Treg cells (14), Th2 cells (20), regulatory B cells (21), and Tr1 cells, the latter of which we focused on in this study. We observed that HDs in the CD46D group produced significantly higher levels of both IFN-γ and IL-10 into cell culture SNs (Figures 7A,B) 60 h post-activation with αCD3/αCD46/IL-2 (Figure 7E). However, the percentage of IFN-γ^+^CD4^+^ and IL-10^+^CD4^+^ T cells measured by flow cytometry was comparable between the two groups (Figures 7C,D), likely due to several factors, including the short incubation time of activated CD4^+^ T cells with Brefeldin A and the different dynamics of cytokine production during final 4 h time incubation period (Figure 7E). Moreover, calcitriol reduced IFN-γ (Figure 7A) while promoted IL-10 (Figure 7B) production in CD4^+^ T cells from HDs CD46D group, whereas CD4^+^ T cells s from the CD46I group produced significantly lower amounts of IFN-γ, that was slightly reduced by calcitriol, but were unable to upregulate IL-10 production after calcitriol stimulation together with αCD3/CD46 (Figures 7A,B). A similar trend was observed in the percentages of IFN-γ^+^CD4^+^ and IL-10^+^CD4^+^ T cells, but with weaker significance (Figures 7C,D). Representative flow cytometry data showing IFN-γ and IL-10 production by CD4^+^ T cells activated with αCD3/αCD46/IL-2 are presented in Figure 7F.

**Figure 8:**
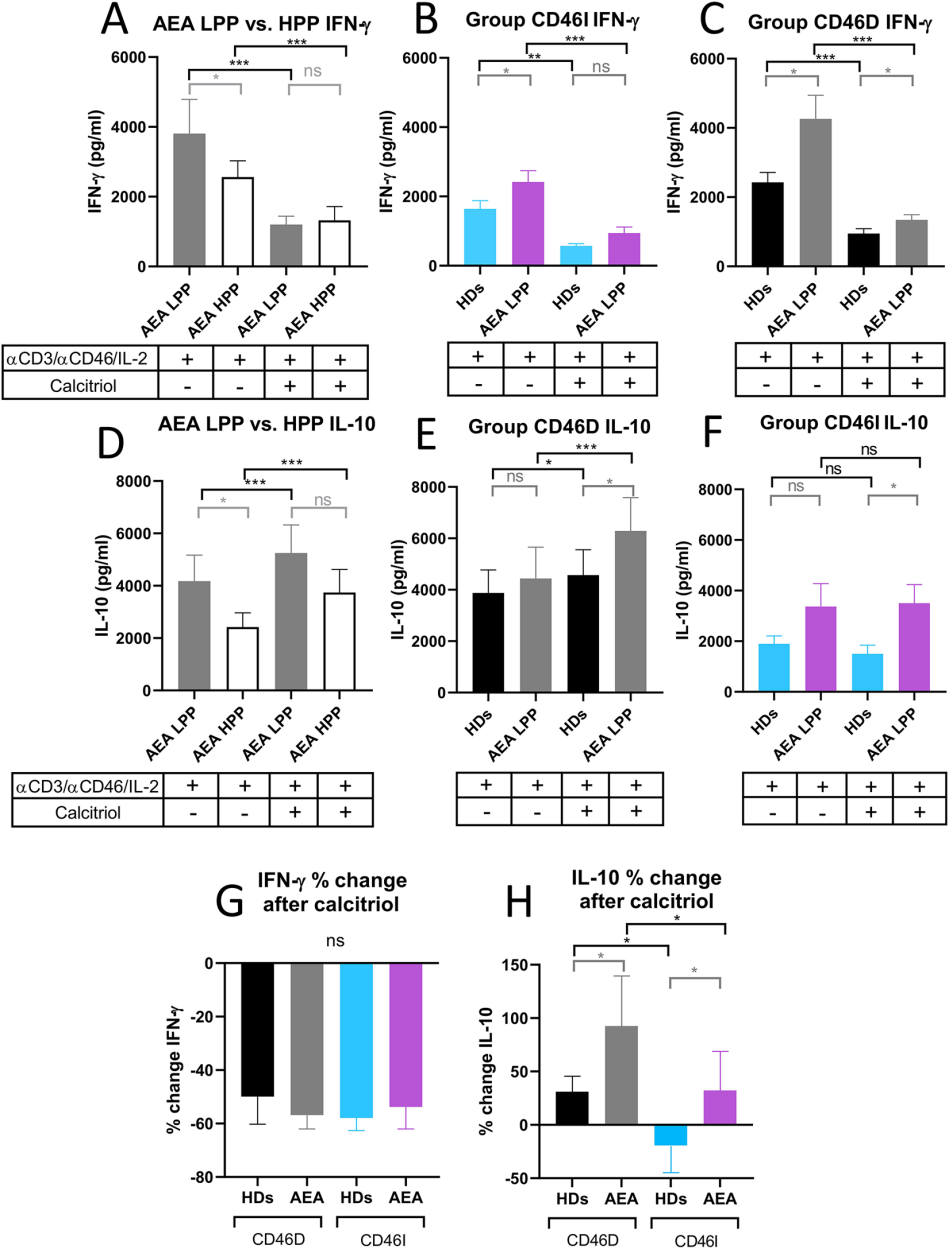
AEA patient's CD4^+^ T cells from CD46D group show increased production of IFN-γ after stimulation, which can be downregulated by calcitriol and simultaneously produce more IL-10. CD4^+^ T cells from 49 HDs and 58 AEA patients were cultured with a mixture of αCD3 (10 μg/ml), αCD46 (5 μg/ml) mAbs and high dose of IL-2 (50 U/ml) (αCD3/*α*CD46/IL-2) or with calcitriol (1 × 10^−7 ^M) (αCD3/αCD46/IL-2/Cal) for 60 h. Concentrations of IFN-γ and IL-10 were measured in cell culture SNs using ELISA. IFN-γ production was compared between AEA LPP and AEA HPP groups **(A)** as well as in groups CD46D **(B)** and CD46I **(C)**. An identical analysis was performed with production of IL-10 **(D–F)**. To simplify the evaluation of the calcitriol's effect in both HDs and AEA patients, with respect to the CD46D/CD46I group division, results are presented as the percentage of downregulation in IFN-γ **(G)** and percentage of upregulation in IL-10 **(H)** after αCD3/αCD46/IL-2/Cal stimulation. Results are presented as the median + 95% CI. Paired data were analyzed using Wilcoxon matched-pairs signed rank test (black color) and unpaired data were analyzed unpaired Mann–Whitney *U*-test (grey color); ns (not significant), *p ≤ 0.05, **p ≤ 0.01, ***p ≤ 0.001. HDs, healthy donors; AEA, allergic eosinophilic asthma; mAbs, monoclonal antibodies; SNs, supernatant; CD46D, group where CD46 expression on CD4^+^ T cells decrease after calcitriol stimulation; CD46I, group where CD46 expression on CD4^+^ T cells increase after calcitriol stimulation; CI, confidence interval.

Next, to provide more insight into modulatory effect of calcitriol on CD4^+^ T cells from AEA patients, we assessed whether AEA patient's CD4^+^ T cells respond differently to αCD3/αCD46/IL-2 stimulation with calcitriol depending on the pollen period. We observed that AEA patients in LPP produced higher levels of both IFN-γ (Figure 8A) and IL-10 (Figure 8D) (IFN-γ median: 3,809 pg/ml, IL-10 median: 4,174 pg/ml) compared to those in HPP (IFN-γ median: 2,562 pg/ml, IL-10 median: 2,277 pg/ml). Nevertheless, calcitriol reduced IFN-γ and promoted IL-10 in CD4^+^ T cells from AEA patients during both pollen seasons, but with a more prominent effect in LPP (IFN-γ median: 1,200 pg/ml, IL-10 median: 5,285 pg/ml) compared to HPP (IFN-γ median: 1,323 pg/ml, IL-10 median: 3,355 pg/ml). Finally, we compared IFN-γ and IL-10 production between the CD46D and CD46I groups. In both studied groups, CD4^+^ T cells from AEA patients produced more IFN-γ (Figures 8B,C) whereas IL-10 was secreted in amounts comparable to those in HDs (Figures 8E,F). However, calcitriol was able to reduce IFN-γ production in AEA patients to the levels close to HDs in both studied groups CD46D (HDs 49%, AEA 57%) and CD46I (HDs 57%, AEA 54%). When we expressed the results as percentage of decrease, there was no difference between the CD46D and CD46I groups in IFN-γ (Figure 8G), but we observed that calcitriol increased IL-10 production in CD4^+^ T cells by 31% in the HDs CD46D group and by 85% in the AEA CD46D group. Additionally, calcitriol decreased IL-10 production in CD4^+^ T cells by 19% in the HDs CD46I group, but conversely increased it by 32% in AEA CD46I group (Figure 8H). In summary, calcitriol promoted a stronger anti-inflammatory response in the CD46D group of HDs, with an even more significant effect in the CD46D group of AEA patients from LPP.

## 4 Discussion

In this study, we analyzed the modulatory effect of calcitriol on the CD46 pathway, focusing on IFN-γ and IL-10 production by CD4^+^ T cells from healthy donors and adult patients with allergic eosinophilic asthma *in vitro*. Molecule CD46 is known for its extensive functions in the immune system (22, 23) which include its role as a crucial costimulatory molecule for human CD4^+^ T cells. Its co-activation with TCR induce the complosome-driven Th1 differentiation with IFN-γ production, which can be eventually switched to IL-10 secreting regulatory Tr1 phenotype (3, 24) depending particularly on cytokine milieu (5). CD46 is therefore able to modulate immune responses to prevent excessive inflammation (25). We observed variable expression of CD46 on CD4^+^ T cells from AEA patients that differed in dependence on pollen season with increased CD46 on both non-activated and stimulated CD4^+^ T cells during the low pollen period (LPP), whereas this phenomenon was absent in HDs. Increased CD46 on non-activated CD4^+^ T cells in LPP AEA was likely not due to a defective shedding, which occurs via matrix metalloproteinases (MMPs) (22) since plasma levels of soluble CD46 were comparable with HDs. Another mechanism which may contribute to CD46 insufficient downregulation after activation is dysregulated process of CD46 internalization, which normally occurs together with MMPs mediated shedding (26). Others have proposed that increased CD46 on CD4^+^ T cells can lower the threshold for T cell activation (22) and may indicate a shift towards a Th1 dominated immune response (27). We observed that CD4^+^ T cells from AEA patients in LPP preferentially differentiate into Th1 phenotype upon activation, indicating its potential involvement in asthma pathophysiology.

In AEA patients, eosinophil counts and serum ECP levels were lower during LPP when compared with HPP. Activated eosinophils are known to influence Th2 polarization in the adaptive immune response through interactions with T cells (28) and production of several pro-inflammatory mediators including ECP (29). Our observation of a negative correlation between serum ECP levels and CD46^+^CD4^+^ T cells suggests that increased eosinophil activation during HPP affects the Th1 response in CD4^+^ T cells, with eosinophil activation being linked to higher Th2 cytokine production (30, 31). Conversely, during LPP, the decrease in ECP levels was associated with upregulated CD46 expression on unstimulated CD4^+^ T cells and the loss of correlations between ECP, CD46, and the percentage of CD46^+^CD4^+^ T cells. These findings align with the well-established mutual suppression of Th1 and Th2 responses (28). The role of the complosome, with CD46 as a central regulatory molecule, has been well documented in the differentiation of effector Th1 cells (5, 7, 24, 25). During Th1 and Th2 differentiation, CD4^+^ T cells upregulate glucose transporter 1 (GLUT1) and other nutrient channels, with CD46 playing a key role (32, 33). In the Th1 response, CD46 activates the protein kinase complex mTORC1, while in the Th2 response, mTORC2 is activated (34). However, it is still unclear whether CD46 also participates in the mTORC2 induction. Given the increased CD46 expression on CD4^+^ T cells and the negative correlation between CD46 and ECP, it is possible that CD46 downregulates during more prominent Th2 response in AEA HPP as a part of the regulation process between Th1 and Th2 responses. This hypothesis, however, requires additional research.

We further investigated how calcitriol modulates CD46 on CD4^+^ T cells from AEA patients. Vitamin D is essential for regulating the immune response and inflammation and vitamin D deficiency is associated with a higher incidence of several diseases, including asthma and multiple sclerosis (MS) (35). Clinical trials have shown mixed results regarding the effectiveness of vitamin D supplementation in the treatment of asthma. Some research suggests that vitamin D supplementation may improve asthma control, reduce exacerbations, and improve lung function, particularly in vitamin D-deficient individuals, depending on individual patient characteristics and baseline vitamin D levels (36). However, other studies have not confirmed these findings (15, 16). *In vitro* effects of calcitriol (hormonal form of vitamin D_3_) on CD4^+^ T cells are well documented: a reduction in CD46 expression and an increase in the number of CD4^+^CD25^+^FoxP3^+^ T cells in 11 patients with MS and 15 healthy controls have been described (19, 37).

In MS patients, calcitriol has been shown to downregulate CD46 expression and modulate T cell responses, affecting the phenotype of CD46-activated T cells (19, 37). Our study involving HDs and patients with AEA shows that there are two different patterns of CD4^+^ T cell response to calcitriol. The majority (approximately 70%; 35/49 HD and 40/58 AEA patients) show a decrease in CD46 expression (CD46D group), similar to findings by Kickler et al. (19). In contrast, a second group (CD46I, approximately 30%; 14/49 HD and 18/58 AEA patients) show an increase in CD46 expression after stimulation with αCD3/αCD46/IL-2 and calcitriol when compared with stimulation αCD3/αCD46/IL-2 alone. Additionally, we found that CD46 expression on CD4^+^ T cells was not dependent on serum 25-OH vitamin D levels in either HDs or AEA patients, even though AEA patients had decreased serum 25-OH vitamin D concentration, which was independent of asthma severity and division into CD46D/I groups. This finding is in concordance with other studies conducted on both adult (38) and pediatric AEA patients (39).

In both CD46D groups comprising HDs and AEA patients, the behavior of CD4^+^ T cells in response to calcitriol was in accordance with the findings mentioned above, specifically the reduction in CD46 expression, increased CD25 expression, decreased IFN-γ production, and increased IL-10 production. Compared to the HDs CD46D group, we observed higher CD46 expression in the AEA CD46D patients, both after αCD3/αCD46/IL-2 stimulation alone or with calcitriol. However, we noticed that calcitriol was able to downregulate CD46 by 40% in CD46D group from HDs, but only by 27% by CD46D group from AEA. This indicates that calcitriol can exert a regulatory effect in AEA CD46D patients, though not to the same extent as in HDs. Therefore, further studies are necessary to determine the cause of calcitriol's limited effect in AEA CD46D patients.

Our data also revealed that αCD3/αCD46/IL-2 stimulation with calcitriol in CD4^+^ T cells increased surface expression of CD25 and also promoted a release of soluble IL-2Rα chain (sIL-2RA) into cell culture SNs, which is in accordance with others (5, 6, 19). However, AEA CD46D patients showed slightly increased CD25 expression at the cell surface after stimulation with αCD3/αCD46/IL-2 and calcitriol. Furthermore, they also released more sIL-2RA into cell culture SNs. However, increased sIL-2RA was found in AEA's bronchoalveolar lavage (BAL) (40) and serum (30) suggesting, that T cells might sustain symptoms in the patients with atopic asthma (40). Interestingly, AEA patients showed reduced proliferation of CD4^+^ T cells when stimulated with αCD3/αCD46/IL-2 regardless of the addition of calcitriol, despite elevated CD25 expression. This may be due to a presence of different ratios of CD4^+^ T cell subsets after *in vitro* stimulation. Notably, the increased percentage of natural CD4^+^CD25^+^ Treg cells is linked to higher CD25 expression and potent IL-10 production that can inhibit the proliferation of effector CD4^+^ T cell subsets in the surrounding milieu (41, 42).

Similarly, IFN-γ production was increased in AEA patients when compared with HDs, both following αCD3/αCD46/IL-2 stimulation alone or with calcitriol addition. We observed that AEA patients produced more IFN-γ after stimulation. Contrary to our results Tsai et al. (43) reported decreased IFN-γ in αCD3/αCD46 stimulated CD4^+^ T cells in response to house dust mites in individuals with asthma. These differences could reflect different AEA cohorts, since we studied adult patients with asthma predominantly allergic to pollens, but Tsai et al. studied small group of pediatric patients allergic to house dust mites. Additionally, the contradictory results may be due to different CD4^+^ T cell subsets within the CD4^+^ T cell pool, since children have a higher proportion of naive T cells, whereas adults have more memory T cells (44). In our cohort of AEA/CD46D patients, increased production of IFN-γ could be downregulated by calcitriol. Furthermore, increased IFN-γ levels correlated with AHR (45) and BAL analysis from AEA patients and revealed a predominance of Th1-associated cytokine secretion profile (46, 47). Additionally, pediatric AEA patients with severe asthma exhibited Th1 and Th17 signatures (46).

To support calcitriol regulatory effect in AEA patients, we also observed a normal IL-10 production by αCD3/αCD46/IL-2 stimulated CD4^+^ T cells, which was significantly upregulated by calcitriol (85% in AEA vs. 31% in HDs) in CD46D group. Contrary to our results, Tsai et al. (43) described decreased IL-10 by *in vitro* stimulated CD4^+^ T cells from pediatric AEA patients. These differences may be from the same reasons as discussed above in the case of IFN-γ. Similarly to our results, calcitriol was able to restore the IL-10/IFN-γ ratio in patients with multiple sclerosis (19) and has been shown to have a similar effect in other autoimmune (5, 48–52) or infectious diseases (53, 54).

However, CD46I group within HDs and AEA patients showed a comparable increase in CD46 expression after stimulation with αCD3/αCD46/IL-2 and calcitriol. This represents an unexpected behavior of CD4^+^ T cells in a relatively large group of individuals: 14/49 HDs and 18/58 AEA patients. Previous studies involving CD4^+^ T cells stimulated with αCD3/αCD46/IL-2 alone (14, 43) or in combination with calcitriol (19) did not include as large cohort of individuals, therefore it is possible that this behavior has not been recognized before. Our data indicate that both AEA/CD46I patients and HDs exhibit reduced reactivity even before calcitriol exposure: they had lower CD46 expression on non-activated CD4^+^ T cells. Furthermore, the HDs/CD46I and AEA/CD46I groups exhibited reduced CD25 expression, and stimulation with calcitriol did not lead to an increase in the number of CD4^+^CD25^+^ T cells in these groups when compared with the CD46D groups. Both CD46I groups exhibited lower IL-10 production after αCD3/αCD46/IL-2 stimulation, which did not induce after calcitriol addition. Moreover, in both CD46I groups, the production of IFN-γ by activated CD4^+^ T cells decreased after exposure to calcitriol, similar to the decrease observed in the CD46D groups after calcitriol exposure. We did not find a similar description of cell behavior in any of the previously studied groups (14, 19, 37, 43). This phenomenon is not related to the age of the patients or controls, as both groups are age-matched. The altered reactivity is present in both healthy controls and patients, indicating that it is not asthma-dependent.

Considering the occurrence of low-responding individuals in both HDs as well as AEA patients, these results may reflect a variability in immunological reactivity across the whole population, particularly in VDR. It has been shown that different genetic polymorphisms in VDR modify the response to vitamin D supplementation (55). Another factor that may have a role is the regulation of VDR expression on cells of the immune system including CD4^+^ T cells after activation, which can be determined not only by genetic factors, but also by environmental factors such as diet, infections, or exposure to sunlight (55, 56). All of these factors might result in a lower state of CD4^+^ T cell activation after calcitriol stimulation in some individuals or may result in delayed switching into Tr1 phenotype thus potentially affecting the efficiency of calcitriol administration in clinical practice.

## 5 Conclusion

Our observations provide new insights into the reactivity of the CD46 molecule on the surface of CD4^+^ T cells. For the first time, we have described the presence of two types of Th1 reactivity in CD4^+^ T cells. These two types of Th1 response in CD4^+^ T cells were termed as groups CD46D and CD46I and were found in both HDs and AEA patients. They differed not only in the level of surface expression of CD46 on CD4^+^ T cells before and after αCD3/αCD46/IL-2 stimulation but also in IFN-γ and IL-10 production upon calcitriol addition with significantly stronger shift towards regulatory phenotype in AEA patients from CD46D group. Thus, the administration of calcitriol *in vivo* may have a promising regulatory effect in individuals with AEA who belong to the CD46D group, potentially when combined with low serum levels of 25-OH vitamin D. Although further research and clinical studies are necessary to confirm these assumptions, our observations provide a new qualitative perspective on the potential mechanisms by which allergic inflammation influences the Th1 response in AEA patients and open new avenues for the therapeutic use of vitamin D, not only in asthmatic patients but also in other diseases.

## Data Availability

The raw data supporting the conclusions of this article will be made available by the authors, without undue reservation.
