# Supplementary material for: Low CD46 expression on activated CD4+ T cells predict improved Th1 cell reactivity to calcitriol in majority of patients with allergic eosinophilic asthma and healthy donors
Source: Front Allergy. 2024 Sep 24;5:1462579. doi: 10.3389/falgy.2024.1462579 (PMC11472004; doi:10.3389/falgy.2024.1462579)
Supplement: Supplementary file 1 [file Datasheet1.pdf]

Supplementary Material

Supplementary Figure 1: AEA patient cohort characteristics based on positivity of specific IgE

The Venn diagram shows the distribution and overlap of major allergens in our AEA patient cohort (n=58), based on positivity of specific IgE, which was measured for pollen, dust mites, animals and molds.

Supplementary Figure 2: Flow cytometry - CD4$^+$ T cell gating strategy

Flow cytometry dot plots depict the gating strategy used to characterize the isolated CD4$^+$ T cells. After the exclusion of doublets (A) and dead cells (B), live CD4$^+$ T cells (D) were divided into four subsets based on their IFN-γ and IL-10 expression (D-F), namely the IFN-γ$^-$ IL-10$^-$, IFN-γ$^+$IL-10$^-$ (Th1), IFN-γ$^-$IL-10$^+$ (Tr1), and IFN-γ$^+$IL-10$^+$ CD4$^+$ T cells. The CD4$^+$ T cells were incubated for 60h without stimuli (non-activated, NA) (D), with αCD3 (10ug/ml), αCD46 (5ug/ml) + IL-2 (50 U/ml) (E), or with αCD3 (10ug/ml), αCD46 (5ug/ml) + IL-2 (50 U/ml) and calcitriol (10^{-7} M) (F). Representative histograms (G, H, I) show the expression of CD46,-CD25 and Ki-67 on NA and stimulated CD4$^+$ T cells. Data are representative sample of a healthy control and were analyzed using the Kaluza software. mAbs (monoclonal antibodies), NA (non-activated), FMO (fluorescence minus one), MFI (median fluorescence intensity).

Supplementary Figure 3: AEA patients exhibit more prominent signs of Th2 response in high pollen period.

(A) Serum concentration of total IgE, ECP, percentage of eosinophils from whole blood and FeNO were measured in AEA patients in both LPP and HPP. Data are presented as median with 95% CI. Statistical analysis was performed using Wilcoxon matched-pairs signed rank test. (B) CD4$^+$ T cells from 58 AEA patients in LPP and in HPP were cultured with a mixture of αCD3 (10 μg/ml), αCD46 (5 μg/ml) mAbs and high dose of IL-2 (50 U/ml) (αCD3/αCD46/IL-2) or with calcitriol ($1*10^{-7}$ M) (αCD3/αCD46/IL-2/Cal) for 60h. Concentrations of sIL-2RA was measured in cell culture SN using ELISA in both pollen periods (LPP vs. HPP). Data from panel B were analyzed using Kruskal-Wallis test with Dunn's correction for multiple comparisons all vs. all; ns (not significant), *$p \leq 0.05$, **$p \leq 0.01$. ECP (eosinophilic cationic protein), FeNO (fractional exhaled nitric oxide), AEA (allergic eosinophilic asthma), LPP (low pollen period), HPP (high pollen period), CI (confidence interval), sIL-2RA (soluble IL-2 α chain).

Supplementary Figure 4: Stimulation affects CD46 expression across the whole population of CD4$^+$ T cells, not only CD4$^+$CD46$^-$ subset.

CD4$^+$ T cells from 49 HDs and 58 patients with AEA were cultivated without stimuli (NA) or with a mixture of αCD3 (10 μg/ml), αCD46 (5 μg/ml) mAbs and high dose of IL-2 (50 U/ml) (αCD3/αCD46/IL-2) or with calcitriol ($1*10^{-7}$ M) (αCD3/αCD46/IL-2/Cal) for 60h. (A) Representative dot plots and histograms depict the changes in CD46 expression on CD4$^+$ T cells under different stimuli, represented as the percentage of positivity and median fluorescence intensity (MFI). (B) MFI and percentage of CD46$^+$CD4$^+$ T cells were analyzed using the Kruskal-Wallis test. Horizontal bar represents the median. ns (not significant), *$p \leq 0.05$, ***$p \leq 0.001$.; HDs (healthy donors), AEA (allergic eosinophilic asthma), mAbs (monoclonal antibodies).
