# Supplementary figures and images for: Low CD46 expression on activated CD4+ T cells predict improved Th1 cell reactivity to calcitriol in majority of patients with allergic eosinophilic asthma and healthy donors
Source: Front Allergy. 2024 Sep 24;5:1462579. doi: 10.3389/falgy.2024.1462579 (PMC11472004; doi:10.3389/falgy.2024.1462579)

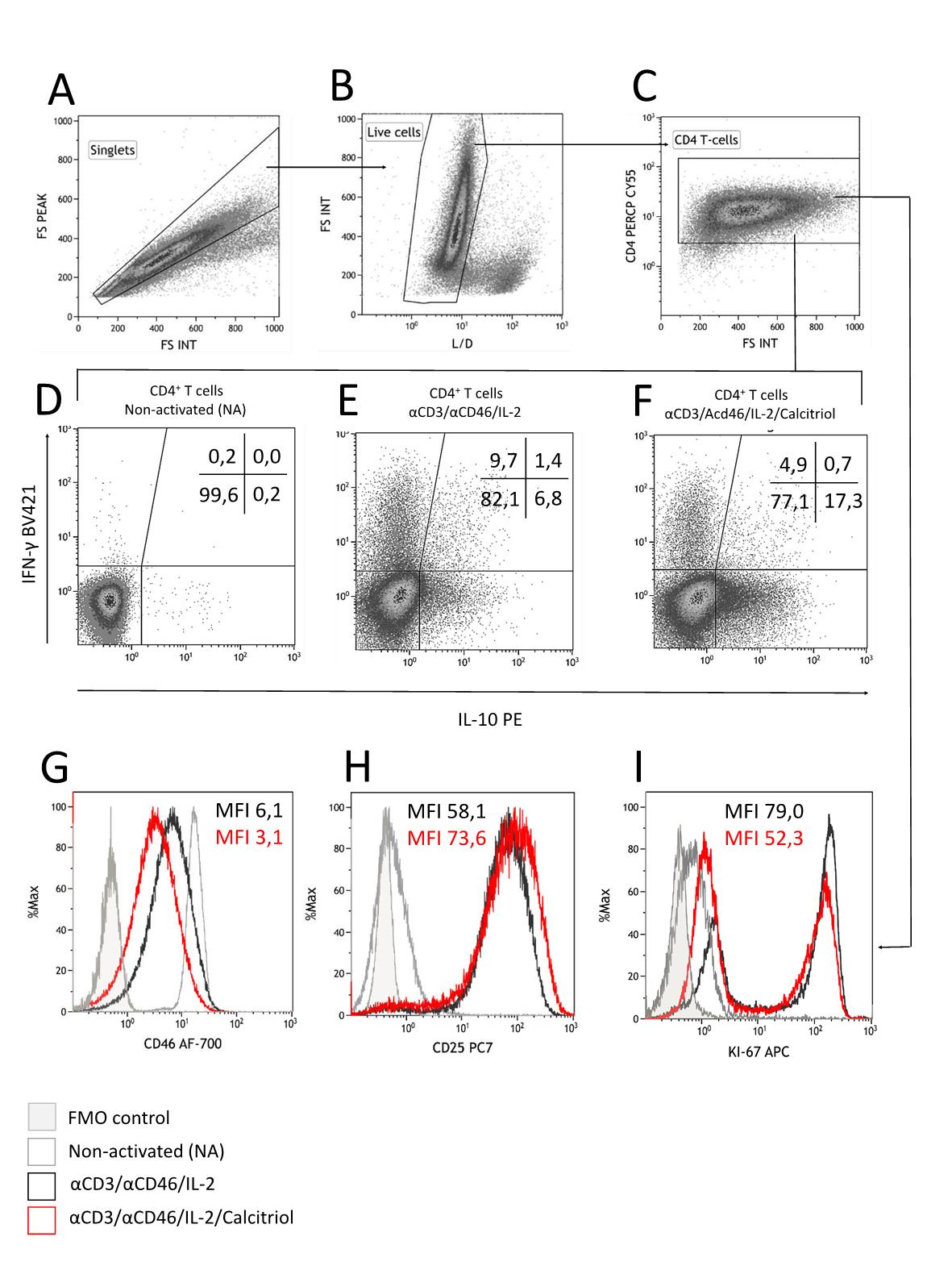

Supplement: Supplementary file 3 [file Image2.jpeg]
